# NLRP3 inflammasome as a sensor of micro- and nanoplastics immunotoxicity

**DOI:** 10.3389/fimmu.2023.1178434

**Published:** 2023-04-18

**Authors:** Andi Alijagic, Alexander Hedbrant, Alexander Persson, Maria Larsson, Magnus Engwall, Eva Särndahl

**Affiliations:** ^1^ Inflammatory Response and Infection Susceptibility Centre (iRiSC), Faculty of Medicine and Health, Örebro University, Örebro, Sweden; ^2^ School of Medical Sciences, Faculty of Medicine and Health, Örebro University, Örebro, Sweden; ^3^ Man-Technology-Environment Research Center (MTM), Örebro University, Örebro, Sweden

**Keywords:** innate immunity, inflammation, plastics, human health, pollution

## Abstract

Micro- and nanoplastics (MNPs) are emerging pollutants with scarcely investigated effects on human innate immunity. If they follow a similar course of action as other, more thoroughly investigated particulates, MNPs may penetrate epithelial barriers, potentially triggering a cascade of signaling events leading to cell damage and inflammation. Inflammasomes are intracellular multiprotein complexes and stimulus-induced sensors critical for mounting inflammatory responses upon recognition of pathogen- or damage-associated molecular patterns. Among these, the NLRP3 inflammasome is the most studied in terms of activation *via* particulates. However, studies delineating the ability of MNPs to affect NLRP3 inflammasome activation are still rare. In this review, we address the issue of MNPs source and fate, highlight the main concepts of inflammasome activation *via* particulates, and explore recent advances in using inflammasome activation for assessment of MNP immunotoxicity. We also discuss the impact of co-exposure and MNP complex chemistry in potential inflammasome activation. Development of robust biological sensors is crucial in order to maximize global efforts to effectively address and mitigate risks that MNPs pose for human health.

## Introduction

**Figure 1:**
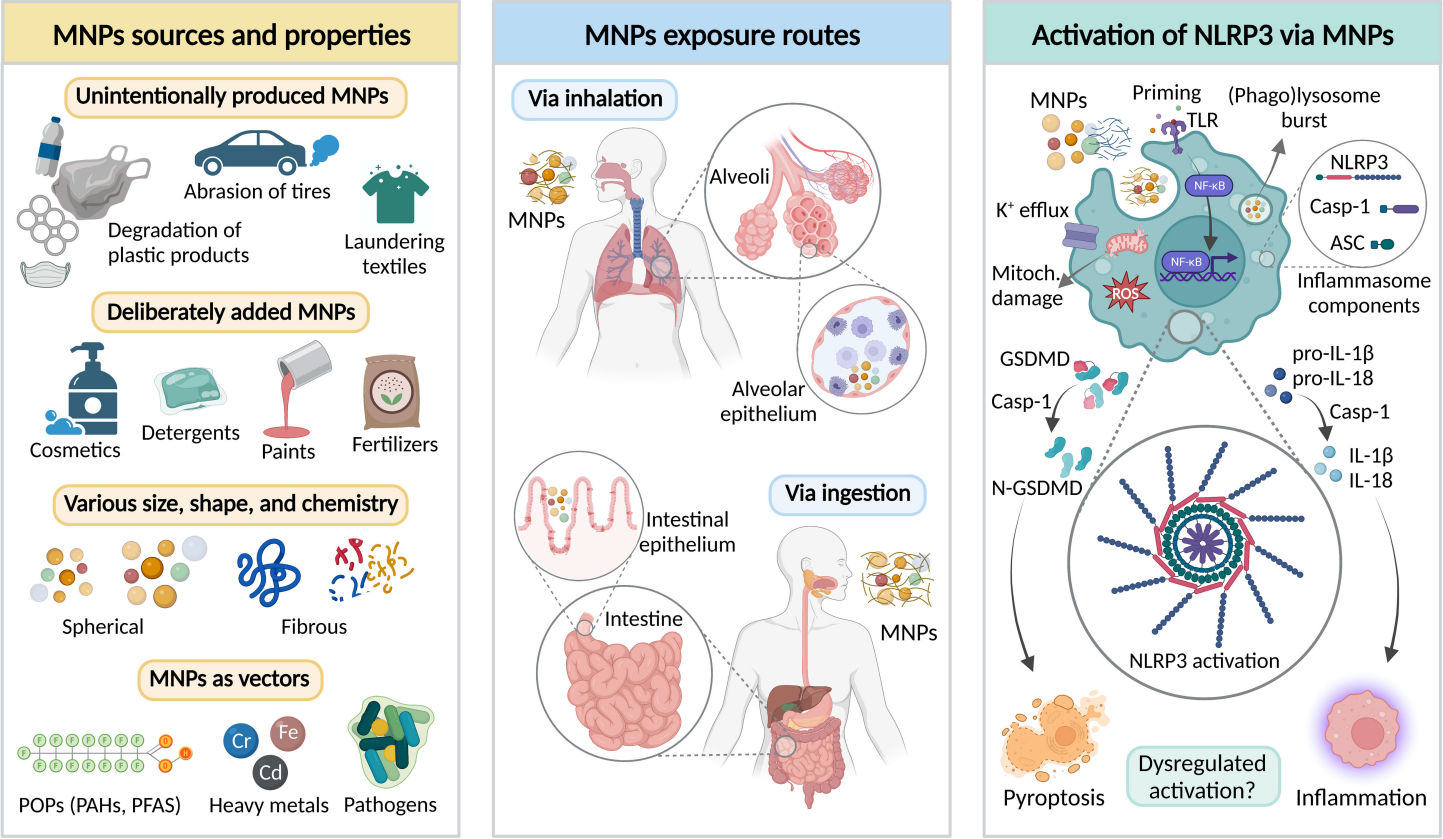
Interplay between micro- and nanoplastics (MNPs) and NLRP3 inflammasome canonical activation pathway. Left panel – major sources and properties of MNPs. MNPs may be unintentionally released or deliberately added to different products. MNPs significantly vary in terms of physicochemical properties, including size, shape, and chemical composition. Importantly, MNPs may act as a vector of various environmental contaminants, such as persistent organic pollutants (POPs), heavy metals, or pathogenic bacteria. PAHs – polycyclic aromatic hydrocarbons; PFAS – Per- and polyfluoroalkyl substances. Middle panel – the main MNP exposure routes in humans include inhalation and ingestion leading to MNP interaction with alveolar and intestinal epithelia. Right panel – Putative mechanisms of MNP-mediated activation of NLRP3 inflammasome in the immunocompetent cells, including Toll-like receptor (TLR)-priming *via* NF-κB resulting in production of NLRP3 inflammasome components, and NLRP3 inflammasome activation leading to the recruitment of the caspase-1 that cleaves its effector substrates, pro-Interleukin-1β (pro-IL-1β), pro-IL-18, and gasdermin-D (GSDMD). The main outcomes of the NLRP3 inflammasome activation include maturation and release of IL-1β and IL-18, and pro-inflammatory cell death (pyroptosis). Figure was created by AA using BioRender.com.

Microplastics and nanoplastics (MNPs) are solid plastic particles at the micro- and nanoscale composed of mixtures of polymers (1). MNPs are a highly diverse class of contaminants, differing in shape (e.g., spherical, fibrous), size, and polymer type; exhibiting a heterogeneity that is typically absent from engineered nanomaterials (2, 3). MNPs found in the environment may additionally contain polymer chemical additives (e.g., plasticizers, stabilizers, colorants, biocidal chemicals), monomers entrapped in the polymer matrix, or adsorbed environmental contaminants (e.g., persistent organic pollutants, heavy metals) (**Figure 1**, left panel) (4–10). As such, MNPs exhibit high environmental mobility, persistence, and low degradation rate. Sources of MNPs are numerous, and they can be unintentionally formed due to e.g., laundering synthetic textiles, abrasion of tires in traffic, degradation of larger plastic objects, etc. Moreover, certain products contain deliberately added MNPs, such as exfoliating beads in facial or body scrubs, fertilizers, plant protection products, detergents, and paints (11–13). A recent body of evidence suggests that humans constantly ingest, inhale, or swallow MNPs after mucociliary clearance (**Figure 1**, middle panel), and the plastics can even be found in the blood, indicating potential of some MNPs to pass the respiratory and intestinal epithelia (14–16). However, if and how MNPs influence human and environmental health is far from being understood.

If MNPs follow a similar course of action as other particulates (e.g., particulate air pollution), they are capable of crossing membranes of epithelia and triggering a cascade of signaling events in the cells, leading to oxidative stress, secretion of cytokines, cellular damage, and inflammation as central common denominators for systemic effects, with subsequent risk at developing cardiovascular and respiratory diseases, allergies, and cancer (1, 17). Several studies have recently described the presence of MNPs in blood, liver, kidney, and even in placenta and brain (18–20). Although the direct biological effect of MNPs in these compartments have not been investigated, it is well known that MNPs are associated with a plethora of chemicals acting as endocrine disruptors and/or genotoxicants. MNPs may also act as vectors carrying opportunistic bacterial pathogens interacting with gut microbiota, thus further impacting host immunity (21–24). The scarce data available on MNP uptake, both *in vivo* and *in vitro*, indicate that only a limited fraction of MNPs is capable of crossing lung and intestinal epithelia. The results show that absorbed fraction *via* intestinal tracts in rodents is low at 0.04–0.3% (25). Moreover, the oral bioavailability level of nano-sized polystyrene is ten to one hundred times greater than the level of micron-sized particles (26, 27). Importantly, MNPs uptake is strongly affected by the formation of biomolecular corona upon entrance in different biological (micro)environments (28). Even if studies indicate low levels of MNP uptake, ubiquitous presence and life-long exposure may lead to accumulation and health-related effects.

Once MNPs arrive at the bio-interface (contact with the cell membranes) or after being internalized, they will encounter the organism’s innate immunity mechanisms, developed for counteracting invading pathogens and for eliminating threatening agents (dust, allergens, dead cells, etc.). Therefore, in order to understand the possible health effects of MNPs, it is important to explore the interaction of MNPs with the innate immune system with an approach capable of evaluating the inflammatory capacity of the interaction (29, 30). One such mechanism is activation of inflammasomes – intracellular multiprotein complexes and stimulus-induced sensors critical for mounting potent pro-inflammatory responses (31). Activation of inflammasome complexes can occur in response to pathogen- or damage-associated molecular patterns (PAMPs or DAMPs), which are signals that inform the host innate immune sensors of a possibly harmful deviation from homeostasis (32).

Four key inflammasomes, namely NLRP1, NLRP3, NLRC4, and AIM2 are described. NLRP1 is extensively expressed in keratinocytes and airway epithelia and recognizes and responds to specific bacteria and diverse pathogen-encoded effectors, including double stranded RNA as well as double stranded DNA (33–35). NLRC4 is mainly associated with innate immune cells and intestinal epithelia and sense several bacterial pathogens and specifically bacterial type III secretion system, and flagellin has been described as potent activators (36). AIM2 responds to pathogen-associated double stranded DNA and is mainly expressed in hematopoietic cells (37, 38). In contrast to the clear pathogen detecting features of these, the nucleotide-binding oligomerization domain (NOD)-like receptor containing pyrin domain 3 (NLRP3) inflammasome (also known as CIAS1, Cryopyrin, NALP3, and Pypaf1) functions rather as a sensor capable of becoming activated following endogenous and exogenous, sterile, and infectious stimuli, as well as environmental pollutants, such as asbestos, silica, or ambient particles (39–41). NLRP3 can be expressed by most cells, and the NLRP3 inflammasome is also activated by a range of nano- and micron-sized particles, e.g., nano-TiO_2_ and nano-SiO_2_ (41–45). Although studies delineating the ability of MNPs to trigger NLRP3 inflammasome activation are rare, given the efficacy of NLRP3 in sensing particulates, inflammasome activation may provide a useful tool for investigations of MNP immunotoxicity. In this review, we discuss the main concepts of inflammasome activation *via* particulates and explore recent advances in using NLRP3 inflammasome activation as an endpoint for the assessment of MNP immunotoxicity.

## NLRP3 inflammasome

The NLRP3 inflammasome is proposed to act as an integrator of different signals arising from the *homeostasis-altering molecular processes* (HAMPS) (46). Therefore, it emerged as a fundamental sensing platform for various PAMPs and DAMPs. Data suggest that NLRP3 inflammasomes can be activated *via* three different pathways 1) canonical NLRP3 inflammasome activation, 2) non-canonical NLRP3 inflammasome activation, and 3) alternative NLRP3 inflammasome activation pathway. Different activation pathways highlight the disparities in NLRP3 activation mechanisms in different cell types and between species.

The canonical activation of the NLRP3 inflammasome is the description of an organized process involving two core steps – priming and activation (**Figure 1**, right panel). Priming is stimulated by exogenous or endogenous molecules (PAMPs or DAMPs) by TLR-mediated signaling cascade leading to NF-κB activation that provides the key components for the later assembly of the NLRP3 inflammasome. (47, 48). The activation step is triggered by ATP, pore-forming toxins, viral RNA, crystals, or particulates leading to the induction of various intracellular events, such as potassium (K^+^) efflux, ROS burst, mitochondrial damage, or (phago)lysosomal rupture that releases the protease cathepsin B (49–55). Upon activation, the NLRP3 inflammasome is assembled by the oligomerization of NLRP3. Afterwards, the pyrin domain of NLRP3 interacts with the apoptosis-associated speck-like protein (ASC) triggering polymerization of ASC to form prion-like filaments, which recruits monomers of the caspase-1 to the NLRP3-ASC oligomer eliciting self-cleavage and activation (56, 57). Subsequently, active caspase-1 proteolytically cleaves and thereby activates pro-IL-1β, pro-IL-18, and gasdermin-D (GSDMD) – providing the plasma membrane pore through which the activated cytokines can be released as well as inducing pyroptosis; the pro-inflammatory form of cell death (58–60).

The non-canonical NLRP3 inflammasome activation, initially described in murine cells, involves caspase-11-mediated signaling, resulting in TLR-independent maturation and release of IL-1β and IL-18, and pyroptotic cell death (61). In humans, the equivalent mechanism is dependent on caspase-4 and caspase-5 (62). These caspases have been described to act as direct receptor molecules for LPS. In addition, upstream involvement of NLRP1 and/or NLRC4 have been proposed, as they can activate caspase-4 and caspase-5 (63, 64). Following this activation mediated by intracellular LPS, these events subsequently lead to K^+^ efflux, which is a central trigger for NLRP3 inflammasome activation and IL-1β release (65, 66). Thus, the NLRP3 inflammasome does become activated but the K^+^ efflux is mediated by involvement of other cellular mechanisms not described in the canonical pathway of inflammasome activation.

The alternative pathway of NLPR3 inflammasome activation is the description of a one-step activation of caspase-1, resulting in IL-1β maturation and secretion (48, 67). In human monocytes, LPS sensing induces a TLR4-TRIF-RIPK1-FADD-CASP8 signaling axis, leading to the cleavage of a yet unidentified caspase-8 substrate that in turn mediates activation of NLRP3 inflammasome (68). Unlike canonical and non-canonical NLRP3 inflammasome activation, the alternative pathway does not require K^+^ efflux and does not induce ASC-speck or pyroptosome formation. The functional biological output is however similar since caspase-1 cleaves and bioactivates pro-IL-1β, which is then released, but the release is however not GSDMD-dependent (69).

In addition to the presence in immunocompetent innate immune cells, an increasing number of studies demonstrate localization and involvement of the NLRP3 inflammasome in cells at important exposure sites, including alveolar, intestinal, and skin epithelia (70–72).

## NLRP3 inflammasome activation by particulates

Particulates found to activate the NLRP3 inflammasome include endogenous particles, such as monosodium urate crystals (MSU) (41), cholesterol crystals (73), fibrillar amyloid-β (74), and fibrillar α-synuclein (75) as well as a large variety of exogenous particles, such as crystalline silica (41), metallic particles (76), fibers, including asbestos (40) and carbon nanotubes (77). Regarding the mechanism of inflammasome activation by particles, several studies have found that particles need to be phagocytosed/endocytosed in order to activate the NLRP3 inflammasome. An exception is crystalline silica particles, where studies have demonstrated both phagocytosis-dependent (41) and -independent (78) NLRP3 inflammasome activation. These contrasting results may be due to differences in the properties of the silica particles, including size, shape, surface properties, or formation of a protein corona coating the particles (79). Following the formation of the phagolysosome, particles may interact with the (phago)lysosomal membrane leading to lysosomal membrane permeabilization (LMP) or rupture with subsequent release of lysosomal content into the cytosol that in turn will activate the NLRP3 inflammasome.

Importantly, release of cathepsin B or NADPH oxidase-generated reactive oxygen species (ROS) are indicated as key mediators of the NLRP3 inflammasome activation, as inhibition of these generally blocks or suppress caspase-1 cleavage and IL-1β release (80, 81). Although a majority of studies demonstrate the role of cathepsin B in inflammasome activation by particles, there are also studies showing the opposite, as summarized by Campden and Zhang (82). It is still not clear how cathepsin B contributes to NLRP3 inflammasome activation, which could depend on actions both related and unrelated of protease activity. Protease unrelated actions in the cytoplasm are suggested by the low enzymatic activity of cathepsins at the neutral cytosolic pH. Cathepsin B has also been found to directly bind the Leucine-Rich Repeat (LRR) domain of NLRP3, and to transiently colocalize with NLRP3 at the endoplasmic reticulum, following treatment with, for example, MSU particles (80). In addition, ROS has been indicated to play a key role in NLRP3 inflammasome activation by particulates in a number of studies (42, 83–86). ROS can be generated by mitochondria but also in phagosomes by the NADPH oxidase NOX2, which is activated, for example, by LPS. ROS-mediated NLRP3 inflammasome activation by asbestos was inhibited when disrupting NOX2, but not when mitochondria-derived ROS was inhibited (40), indicating an important role of NADPH oxidase (NOX)-dependent ROS production in asbestos-induced NLRP3 inflammasome activation. Moreover, Bauernfeind et al. (87) showed that ROS inhibitors interfere with the priming step that is required to induce NLRP3 expression, whereas ROS inhibition does not affect direct NLRP3 activation when NLRP3 is constitutively expressed.

Of note, K^+^ efflux seems to be a common mechanism of NLRP3 inflammasome activation for all known triggers of the inflammasome, including particles (74, 78, 88), but the link between cathepsin B release, ROS, and K^+^ efflux is so far obscure.

## NLRP3 inflammasome activation by micro- and nanoplastics

If MNPs follow a similar course of action as other particulates, they may penetrate epithelial barriers, interact with various cell types, and trigger a number of signaling pathways, including NLRP3 activation. However, studies focusing on the ability of MNPs to activate the NLRP3 inflammasome are still limited. An overview of those, both *in vitro* and *in vivo* studies, is given in **Table 1**. The idea of studying NLRP3 inflammasome activation by MNPs is rather recent as most papers on the topic were published over the last two years.

**Table 1:** Overview of the studies, identified and included in the present review, describing impact of micro- and nanoplastics (MNPs) on the NLRP3 inflammasome activation both *in vitro* and *in vivo*.

Experimental model	Type and size of MNPs	Dose of MNPs	Exposure route	Note on impact	Reference
Monocyte-derived human macrophages (*in vitro*)	Amino- (115 ± 9 nm), carboxyl- (119 ± 7 nm), and non-functionalized (119 ± 5 nm) polystyrene particles	100 μg/mL	Direct deposition on cells	Only amino-functionalized polystyrene induced lysosomal rupture, ROS accumulation, and activated NLRP3 inflammasome	89
Human monocytic cells - THP-1 (*in vitro*)	Amino-functionalized polystyrene (50 nm), polystyrene (50 nm), polyvinyl chloride (235 nm), polyethylene (0.61 µm), polyethylene terephthalate (16 nm and 5.7 µm), polyester fibers (18.5 µm x 10 µm), polyamide 6 fibers (27.5 µm x 10 µm)	50 µg/cm^2^	Direct deposition on cells	Only amino-functionalized polystyrene acted as a direct NLRP3 activator, as seen by increase in IL-1β levels	90
Human gingival fibroblasts (hGFs) (*in vitro*)	MNPs from the Adriatic Sea; 100 nm particles (1 m depth), 0.6 µm particles (24 m depth), (100 nm particles (78 m depth)	Unspecified concentration	Direct deposition on cells	Increased levels of inflammatory markers NF-κB, MyD88 and NLRP3 in terms of proteins and gene expression	91
Human embryonic kidney cells - HEK293 (*in vitro*)	Polystyrene particles (3.39 ± 0.30 µm)	3 - 300 ng/mL	Direct deposition on cells	Exposure reduced NLRP3 protein level	92
Mouse lung epithelial cells - MLE-12 (*in vitro*)	Amino-functionalized polystyrene particles (100 nm)	12.5 μg/mL	Direct deposition on cells	Induced NLRP3/caspase-1 signaling pathway and GSDMD cleavage	93
Mouse hepatocyte cells alpha mouse liver 12 - AML12 (*in vitro*)	Polystyrene particles (~100 nm)	100 μg/mL	Direct deposition on cells	Increased gene and protein expression of NLRP3 and caspase-1	94
Rat (*in vivo*)	Polystyrene particles (0.5 µm)	0.015 - 1.5 mg/kg/day	Drinking water	Pyroptosis and apoptosis of ovarian granulosa cells *via* NLRP3/caspase-1 signaling pathway	95
Rat (*in vivo*)	Polystyrene particles (0.51 µm)	0.5 - 50 mg/L	Drinking water	Activation of NLRP3/caspase-1 signaling pathway and pyroptosis in cardiomyocytes	96
Mouse (*in vivo*)	Polystyrene particles (0.5 μm)	10 - 100 μg/mL	Oral administration	Increased NLRP3, ASC, and cleaved caspase-1 protein levels in the mid colon	97
Mouse (*in vivo*)	Polystyrene particles (5 µm)	0.1-1 mg/mL	Intragastrical instillation	Activation of NLRP3 leading to pyroptosis and ferroptosis in liver cells	98
Mouse (*in vivo*)	Polystyrene particles (~100 nm)	5 μg/g·body weight	Intraperitoneal injection	Increased expression of NLRP3 and maturation of IL-1β in liver tissue	94
Mouse (*in vivo*)	Polystyrene, polypropylene, and polyvinyl chloride particles (size unspecified)	5 mg/mL	Intratracheal instillation	Polystyrene and polypropylene particles increased the protein levels of the NLRP3 components in the lung tissue	99
Mouse (*in vivo*)	Polystyrene particles (0.5 µm), and mixture of polystyrene and arsenic (As)	0.5 ppm polystyrene, and mixture of 0.5 ppm polystyrene + 5 ppm As	Drinking water	Co-exposure to polystyrene and As induced liver pyroptosis by activating the NLRP3/caspase-1 signaling pathway	100
Mouse (*in vivo*)	Polystyrene particles (100 nm), and mixture of polystyrene and LPS	5 μg/g polystyrene, and 5 μg/g polystyrene + 20 μg/g LPS	Intraperitoneal injection	Polystyrene deteriorate LPS-modulated duodenal permeability and inflammation *via* ROS driven-NF-κB/NLRP3 pathway	101
Mouse (*in vivo*)	Carboxylate-functionalized polystyrene particles (mixture of 40 nm and 0.2 µm)	0.01 - 0.1 mg/day	Oral administration	Increase of NLRP3 gene expression in hippocampal samples of female mice; opposite effect in males.	102
Mouse (*in vivo*)	Amino-functionalized polystyrene particles (100 nm)	5 mg/kg	Intratracheal instillation	Activated NLRP3 signaling pathway to drive cleavage of GSDMD and pyroptosis in lung tissue	93
Chicken (*in vivo*)	Polystyrene particles (5 µm)	1-10 mg/L	Drinking water	Cardiac pyroptosis and inflammation by the NF-κB-NLRP3-GSDMD axis	103
Chicken (*in vivo*)	Polystyrene particles (5 μm)	1 -100 mg/L	Drinking water	Activated the ASC-NLRP3-GSDMD signaling pathway and the release of IL-18 and IL-1β in the brain tissue	104
Carp (*in vivo*)	Polyethylene particles (8 μm)	1 µg/L	Gills-mediated uptake	Apoptosis in gills due to the activation of NF-κB/NLRP3 pathway	105

### *In vitro* studies

Polystyrene is to date the most studied type of MNPs in terms of NLRP3 activation. A rare study on human primary macrophages, focusing on MNPs and inflammasome signaling, revealed that amino-modified polystyrene particles (115 ± 9 nm) induced ROS accumulation and NLRP3 inflammasome activation (89). Moreover, the same study disclosed that scavenging of ROS abolished the NLRP3 inflammasome activation. *In vitro* studies on both human monocytic (THP-1) and mouse lung (MLE-12) cell lines outlined the ability of amino-modified polystyrene particles (50-100 nm) to induce activation of the NLRP3/caspase-1 signaling pathway leading to maturation of IL-1β and cleavage of GSDMD (90, 93). Similarly, Chi et al. (94) demonstrated that even non-functionalized polystyrene particles (~100 nm) induced ROS and increased gene and protein expression of NLRP3 and caspase-1 in mouse hepatocytes (AML12 cell line). Interestingly, they found that inhibition of NLRP3 could alleviate the production of ROS induced by exposure to polystyrene particles. In addition, micron-sized polystyrene particles have been found to decrease NLRP3 protein levels in human embryonic kidney cells (HEK293) (92). Taken together, these findings suggest that size and/or functionalization of polystyrene MNPs as well as the cell model utilized, play a major role for the interpretation and involvement of NLRP3 signaling.

### *In vivo* studies

*In vivo* studies disclosing the interplay between MNPs and NLRP3 inflammasome signaling have been conducted on mice (7), rats (2), birds (2), and fish (1), as summarized in **Table 1**. Most studies have examined exposure through the gastrointestinal system, i.e., *via* drinking water, oral or intragastric administration. In addition, some studies have performed intraperitoneal or intratracheal administration, or studied gill-mediated uptake in fish. The studies conducted on rats, exposed to micron-sized polystyrene particles, disclosed activation of the NLRP3/caspase-1 signaling pathway leading to pyroptosis (95, 96). Several studies performed on murine models also revealed activation of the NLRP3/caspase-1 signaling pathway, promoting inflammatory responses, and in several cases MNP exposure led to pyroptosis (93, 94, 97–99, 102). However, NLRP3 activation and pyroptosis were mainly assumed based on the gene or protein expression, lacking more detailed mechanistic description and confirmation e.g., by using NLRP3 knockout models.

Even if the studies conducted on rats and mice analyzed a number of different cell/tissue samples, it is hard to draw conclusions on similarity/differences in responses due to the variable routes of exposure as well as different size and exposure concentrations of MNPs. Still, ROS/oxidative stress seems to act as the common event upstream of the NLRP3 inflammasome machinery governing its activation by MNPs (95, 96, 98, 103). That is not surprising, since ROS constitute one of the most conserved danger signals that are generated after particle phagocytosis (40).

### Co-exposure and leaching studies

Organisms in a polluted environment are generally exposed to mixtures of MNPs, chemical contaminants, and/or pathogens, making it hard to extrapolate what component(s) drive(s) toxicity. However, co-exposure studies focusing on NLRP3 inflammasome activation by exposure of cells or organisms to the mixtures of MNPs, or MNPs plus other contaminants, are still in their infancy. In a recent study, Nikolic et al. (102) demonstrated that a mixture of micron and nano-sized carboxylate-modified polystyrene particles induced increased NLRP3 gene expression in hippocampal samples of female mice. Interestingly, the results were the opposite when conducted in male mice; data outlining that biological sex may play a major role in the NLRP3-mediated response to MNPs. Moreover, a study by Zhong et al. (100) demonstrated that polystyrene exposure with arsenic (As) activated NLRP3/caspase-1 signaling and liver pyroptosis in mice. He et al. (101) showed that polystyrene particles deteriorate LPS-modulated duodenal permeability and trigger inflammation *via* ROS and the NF-κB/NLRP3 pathway. Even if rare, these studies disclose that different contaminants or pathogens modulate MNP-mediated NLRP3 activation.

For the scope of this article, we were unable to identify studies dealing with NLRP3 inflammasome activation *via* leaching of residual monomers or chemical additives present in MNPs. However, our preliminary data, investigating plastics from electronic waste, disclosed that plastics-associated chemicals may lead to inflammatory responses involving NLRP3 inflammasome, as we observed increased secretion of IL-1β from exposed THP-1 cells (unpublished data). However, our observation and the aspects of inflammasome activation due to leaching of chemicals from MNPs require further mechanistic research.

### Limitations of the existing studies

All together, several limitations of the existing studies should be highlighted and taken as a guide when designing and harmonizing future research on MNPs and NLRP3 inflammasome interplay, similarly to the previous and ongoing nanosafety research (106). Major limitations include unclear experimental design, unspecified size of particles, unspecified concentrations, nonuniform concentration reporting, or undefined characterization approaches. All this makes it difficult to compare effects of similar and/or different types of MNPs on NLRP3 inflammasome activation across different studies. Moreover, the most common exposure route was *via* drinking water, however, by that approach it is impossible to precisely determine the amount of ingested MNPs as numerous factors affect water intake during the exposure period. In addition, the majority of the studies were conducted by using polystyrene MNPs, while other MNPs such as polypropylene, polyethylene, and polyethylene terephthalate are the main polymeric materials found in the environment (107). Therefore, it is of critical importance that future research, focusing on NLRP3 inflammasome activation, also considers the great diversity of MNPs found in the environment. It is also important to emphasize the need, in experimental studies, to “coat” MNPs with the relevant biomolecular corona (MNPs never interact with cells as pristine particles), to use realistic MNP concentrations, and to perform longitudinal studies (to assess whether inflammasome activation becomes persistent or not). In addition, novel approaches in the nanosafety field, such as high-content screening combined with multi-omics (108), may be helpful tools in dissecting the cellular and molecular phenotypes upon inflammasome activation by MNPs.

## Conclusions

In the context of the ubiquitous presence and potentially life-long human exposure to MNPs, development of robust biological sensors is crucial in order to maximize global efforts to effectively quantify and mitigate risks that MNPs pose for the human and environmental health. NLRP3 activation and regulation are critical for the host defense. On the quest for new sensors of MNP immunotoxicity, looking towards NLRP3 inflammasome could provide new perspectives, however the field is still in its infancy. As recently highlighted by Yang et al. (109), MNPs may affect immune system in a number of ways, including activation of the inflammasome. However, it is important to emphasize that inflammasome activation does not mean toxicity *per se*, it means activation of a defensive response, which only in a few cases (e.g., anomalous or chronic inflammation) may become damaging to the organism. Cell death is part of such defensive response and, at the level of the whole organism, the death of some immune cells during a defensive response is inconsequential (29). In this review, we have explored recent advances in using NLRP3 inflammasome activation as a potentially important and sensitive readout for the assessment of MNP immunotoxicity, discussed major limitations of the existing studies, and emphasized the need to develop harmonized experimental designs that will ensure comparison of MNP-mediated effects on NLRP3 activation across studies. Upcoming research will further illuminate the potential of the NLRP3 inflammasome to act as a sensor of MNP immunotoxicity.

## Author contributions 

AA conceived the concept, wrote the manuscript, and prepared figure. AH wrote section NLRP3 inflammasome activation by particulates. All authors provided major contributions in the interpretation of knowledge and manuscript revision, and approved the submitted version.

## References

[1] Vethaak AD Legler J. Microplastics and human health. Science (2021) 371(6530):672–4. doi: 10.1126/science.abe5041 33574197

[2] Rochman CM Brookson C Bikker J Djuric N Earn A Bucci K. Rethinking microplastics as a diverse contaminant suite. Environ Toxicol Chem (2019) 38(4):703–11. doi: 10.1002/etc.4371 30909321

[3] Gigault J El Hadri H Nguyen B Grassl B Rowenczyk L Tufenkji N. Nanoplastics are neither microplastics nor engineered nanoparticles. Nat nanotechnol (2021) 16(5):501–7. doi: 10.1038/s41565-021-00886-4 33927364

[4] Meeker JD Sathyanarayana S Swan SH. Phthalates and other additives in plastics: human exposure and associated health outcomes. Philos Trans R Soc B: Biol Sci (2009) 364(1526):2097–113. doi: 10.1098/rstb.2008.0268 PMC2873014 19528058

[5] Teuten EL Saquing JM Knappe DR Barlaz MA Jonsson S Björn A. Transport and release of chemicals from plastics to the environment and to wildlife. Philos Trans R Soc B: Biol Sci (2009) 364(1526):2027–45. doi: 10.1098/rstb.2008.0284 PMC2873017 19528054

[6] Velzeboer I Kwadijk CJAF Koelmans AA. Strong sorption of PCBs to nanoplastics, microplastics, carbon nanotubes, and fullerenes. Environ Sci Technol (2014) 48(9):4869–76. doi: 10.1021/es405721v 24689832

[7] Brennecke D Duarte B Paiva F Caçador I Canning-Clode J. Microplastics as vector for heavy metal contamination from the marine environment. Estuarine Coast Shelf Sci (2016) 178:189–95. doi: 10.1016/j.ecss.2015.12.003

[8] Hahladakis JN Velis CA Weber R Iacovidou E Purnell P. An overview of chemical additives present in plastics: Migration, release, fate and environmental impact during their use, disposal and recycling. J hazard mater (2018) 344:179–99. doi: 10.1016/j.jhazmat.2017.10.014 29035713

[9] Rodrigues JP Duarte AC Santos-Echeandía J Rocha-Santos T. Significance of interactions between microplastics and POPs in the marine environment: a critical overview. TrAC Trends Anal Chem (2019) 111:252–60. doi: 10.1016/j.trac.2018.11.038

[10] Zimmermann L Dierkes G Ternes TA Völker C Wagner M. Benchmarking the *in vitro* toxicity and chemical composition of plastic consumer products. Environ Sci Technol (2019) 53(19):11467–77. doi: 10.1021/acs.est.9b02293 31380625

[11] Wright SL Kelly FJ. Plastic and human health: a micro issue? Environ Sci Technol (2017) 51(12):6634–47. doi: 10.1021/acs.est.7b00423 28531345

[12] Stapleton PA. Microplastic and nanoplastic transfer, accumulation, and toxicity in humans. Curr Opin Toxicol (2021) 28:62–9. doi: 10.1016/j.cotox.2021.10.001 PMC8654083 34901583

[13] SAPEA. A scientific perspective on microplastics in nature and society. (2019). doi: 10.26356/microplastics

[14] Catarino AI Macchia V Sanderson WG Thompson RC Henry TB. Low levels of microplastics (MP) in wild mussels indicate that MP ingestion by humans is minimal compared to exposure *via* household fibres fallout during a meal. Environ pollut (2018) 237:675–84. doi: 10.1016/j.envpol.2018.02.069 29604577

[15] Koelmans AA Nor NHM Hermsen E Kooi M Mintenig SM De France J. Microplastics in freshwaters and drinking water: Critical review and assessment of data quality. Water Res (2019) 155:410–22. doi: 10.1016/j.watres.2019.02.054 PMC6449537 30861380

[16] Wright SL Ulke J Font A Chan KLA Kelly FJ. Atmospheric microplastic deposition in an urban environment and an evaluation of transport. Environ Int (2020) 136:105411. doi: 10.1016/j.envint.2019.105411 31889555 PMC7013824

[17] Kelly FJ Fussell JC. Toxicity of airborne particles–established evidence, knowledge gaps and emerging areas of importance. Philos Trans R Soc A (2020) 378(2183):20190322. doi: 10.1098/rsta.2019.0322 PMC7536031 32981440

[18] Gruber MM Hirschmugl B Berger N Holter M Radulović S Leitinger G. Plasma proteins facilitates placental transfer of polystyrene particles. J Nanobiotechnol (2020) 18(1):1–14. doi: 10.1186/s12951-020-00676-5 PMC7487953 32907583

[19] Prüst M Meijer J Westerink RH. The plastic brain: neurotoxicity of micro-and nanoplastics. Particle fibre Toxicol (2020) 17(1):1–16. doi: 10.1186/s12989-020-00358-y PMC7282048 32513186

[20] Yong CQY Valiyaveettil S Tang BL. Toxicity of microplastics and nanoplastics in mammalian systems. Int J Environ Res Public Health (2020) 17(05):1509. doi: 10.3390/ijerph17051509 32111046 PMC7084551

[21] Muncke J. Exposure to endocrine disrupting compounds *via* the food chain: Is packaging a relevant source? Sci total Environ (2009) 407(16):4549–59. doi: 10.1016/j.scitotenv.2009.05.006 19482336

[22] Bang DY Kyung M Kim MJ Jung BY Cho MC Choi SM. Human risk assessment of endocrine-disrupting chemicals derived from plastic food containers. Compr Rev Food Sci Food Saf (2012) 11(5):453–70. doi: 10.1111/j.1541-4337.2012.00197.x

[23] Lu L Luo T Zhao Y Cai C Fu Z Jin Y. Interaction between microplastics and microorganism as well as gut microbiota: A consideration on environmental animal and human health. Sci Total Environ (2019) 667:94–100. doi: 10.1016/j.scitotenv.2019.02.380 30826685

[24] Muncke J. Tackling the toxics in plastics packaging. PloS Biol (2021) 19(3):e3000961. doi: 10.1371/journal.pbio.3000961 33784315 PMC8009362

[25] Carr KE Smyth SH McCullough MT Morris JF Moyes SM. Morphological aspects of interactions between microparticles and mammalian cells: intestinal uptake and onward movement. Prog Histochem Cytochem (2012) 46(4):185–252. doi: 10.1016/j.proghi.2011.11.001 22240063

[26] Jani P Halbert GW Langridge J Florence AT. Nanoparticle uptake by the rat gastrointestinal mucosa: quantitation and particle size dependency. J Pharm Pharmacol (1990) 42(12):821–6. doi: 10.1111/j.2042-7158.1990.tb07033.x 1983142

[27] Walczak AP Kramer E Hendriksen PJ Tromp P Helsper JP van der Zande M. Translocation of differently sized and charged polystyrene nanoparticles in *in vitro* intestinal cell models of increasing complexity. Nanotoxicology (2015) 9(4):453–61. doi: 10.3109/17435390.2014.944599 25093449

[28] Tenzer S Docter D Kuharev J Musyanovych A Fetz V Hecht R. Rapid formation of plasma protein corona critically affects nanoparticle pathophysiology. In: Nano-enabled medical applications. Jenny Stanford Publishing (2020). p. 251–78. 10.1038/nnano.2013.181 24056901

[29] Boraschi D Alijagic A Auguste M Barbero F Ferrari E Hernadi S. Addressing nanomaterial immunosafety by evaluating innate immunity across living species. Small (2020) 16(21):2000598. doi: 10.1002/smll.202000598 32363795

[30] Boraschi D Canesi L Drobne D Kemmerling B Pinsino A Prochazkova P. Interaction between nanomaterials and the innate immune system across evolution. Biol Rev (2023). doi: 10.1111/brv.12928 36639936

[31] Martinon F Burns K Tschopp J. The inflammasome: a molecular platform triggering activation of inflammatory caspases and processing of proIL-β. Mol Cell (2002) 10(2):417–26. doi: 10.1016/S1097-2765(02)00599-3 12191486

[32] Próchnicki T Latz E. Inflammasomes on the crossroads of innate immune recognition and metabolic control. Cell Metab (2017) 26(1):71–93. doi: 10.1016/j.cmet.2017.06.018 28683296

[33] Neiman-Zenevich J Stuart S Abdel-Nour M Girardin SE Mogridge J. Listeria monocytogenes and shigella flexneri activate the NLRP1B inflammasome. Infect Immun (2017) 85(11):e00338–17. doi: 10.1128/IAI.00338-17 PMC5649023 28808162

[34] Bauernfried S Scherr MJ Pichlmair A Duderstadt KE Hornung V. Human NLRP1 is a sensor for double-stranded RNA. Science (2021) 371(6528):eabd0811. doi: 10.1126/science.abd0811 33243852

[35] Zhou JY Sarkar MK Okamura K Harris JE Gudjonsson JE Fitzgerald KA. Activation of the NLRP1 inflammasome in human keratinocytes by the dsDNA mimetic poly (dA: dT). Proc Natl Acad Sci (2023) 120(5):e2213777120. doi: 10.1073/pnas.2213777120 36693106 PMC9945980

[36] Wen J Xuan B Liu Y Wang L He L Meng X. Updating the NLRC4 inflammasome: from bacterial infections to autoimmunity and cancer. Front Immunol (2021) 12:702527. doi: 10.3389/fimmu.2021.702527 34276697 PMC8283967

[37] Hornung V Ablasser A Charrel-Dennis M Bauernfeind F Horvath G Caffrey DR. AIM2 recognizes cytosolic dsDNA and forms a caspase-1-activating inflammasome with ASC. Nature (2009) 458(7237):514–8. doi: 10.1038/nature07725 PMC2726264 19158675

[38] Kaminski JJ Schattgen SA Tzeng TC Bode C Klinman DM Fitzgerald KA. Synthetic oligodeoxynucleotides containing suppressive TTAGGG motifs inhibit AIM2 inflammasome activation. J Immunol (2013) 191(7):3876–3883. 23986531 10.4049/jimmunol.1300530 PMC3878640

[39] Martinon F Agostini L Meylan E Tschopp J. Identification of bacterial muramyl dipeptide as activator of the NALP3/cryopyrin inflammasome. Curr Biol (2004) 14(21):1929–34. doi: 10.1016/j.cub.2004.10.027 15530394

[40] Dostert C Pétrilli V Van Bruggen R Steele C Mossman BT Tschopp J. Innate immune activation through Nalp3 inflammasome sensing of asbestos and silica. Science (2008) 320(5876):674–7. doi: 10.1126/science.1156995 PMC2396588 18403674

[41] Hornung V Bauernfeind F Halle A Samstad EO Kono H Rock KL. Silica crystals and aluminum salts activate the NALP3 inflammasome through phagosomal destabilization. Nat Immunol (2008) 9(8):847–56. doi: 10.1038/ni.1631 PMC2834784 18604214

[42] Yazdi AS Guarda G Riteau N Drexler SK Tardivel A Couillin I. Nanoparticles activate the NLR pyrin domain containing 3 (Nlrp3) inflammasome and cause pulmonary inflammation through release of IL-1α and IL-1β. Proc Natl Acad Sci (2010) 107(45):19449–54. doi: 10.1073/pnas.1008155107 PMC2984140 20974980

[43] Baron L Gombault A Fanny M Villeret B Savigny F Guillou N. The NLRP3 inflammasome is activated by nanoparticles through ATP, ADP and adenosine. Cell Death Dis (2015) 6(2):e1629. doi: 10.1038/cddis.2014.576 25654762 PMC4669808

[44] Sharma B McLeland CB Potter TM Stern ST Adiseshaiah PP. Assessing NLRP3 inflammasome activation by nanoparticles. Characterization Nanoparticles Intended Drug Delivery (2018) 1682:135–47. doi: 10.1007/978-1-4939-7352-1_12 29039099

[45] Shirasuna K Karasawa T Takahashi M. Exogenous nanoparticles and endogenous crystalline molecules as danger signals for the NLRP3 inflammasomes. J Cell Physiol (2019) 234(5):5436–50. doi: 10.1002/jcp.27475 30370619

[46] Liston A Masters SL. Homeostasis-altering molecular processes as mechanisms of inflammasome activation. Nat Rev Immunol (2017) 17(3):208–14. doi: 10.1038/nri.2016.151 28163301

[47] Bauernfeind FG Horvath G Stutz A Alnemri ES MacDonald K Speert D. Cutting edge: NF-κB activating pattern recognition and cytokine receptors license NLRP3 inflammasome activation by regulating NLRP3 expression. J Immunol (2009) 183(2):787–91. doi: 10.4049/jimmunol.0901363 PMC2824855 19570822

[48] Netea MG Nold-Petry CA Nold MF Joosten LA Opitz B van der Meer JH. Differential requirement for the activation of the inflammasome for processing and release of IL-1β in monocytes and macrophages. Blood (2009) 113(10):2324–35. doi: 10.1182/blood-2008-03-146720 PMC2652374 19104081

[49] Perregaux D Gabel CA. Interleukin-1 beta maturation and release in response to ATP and nigericin. evidence that potassium depletion mediated by these agents is a necessary and common feature of their activity. J Biol Chem (1994) 269(21):15195–203. doi: 10.1016/S0021-9258(17)36591-2 8195155

[50] Zhou R Yazdi AS Menu P Tschopp J. A role for mitochondria in NLRP3 inflammasome activation. Nature (2011) 469(7329):221–5. doi: 10.1038/nature09663 21124315

[51] Lee GS Subramanian N Kim AI Aksentijevich I Goldbach-Mansky R Sacks DB. The calcium-sensing receptor regulates the NLRP3 inflammasome through Ca2+ and cAMP. Nature (2012) 492(7427):123–7. doi: 10.1038/nature11588 PMC4175565 23143333

[52] Shimada K Crother TR Karlin J Dagvadorj J Chiba N Chen S. Oxidized mitochondrial DNA activates the NLRP3 inflammasome during apoptosis. Immunity (2012) 36(3):401–14. doi: 10.1016/j.immuni.2012.01.009 PMC3312986 22342844

[53] Iyer SS He Q Janczy JR Elliott EI Zhong Z Olivier AK. Mitochondrial cardiolipin is required for Nlrp3 inflammasome activation. Immunity (2013) 39(2):311–23. doi: 10.1016/j.immuni.2013.08.001 PMC3779285 23954133

[54] Muñoz-Planillo R Kuffa P Martínez-Colón G Smith BL Rajendiran TM Núñez G. K+ efflux is the common trigger of NLRP3 inflammasome activation by bacterial toxins and particulate matter. Immunity (2013) 38(6):1142–53. doi: 10.1016/j.immuni.2013.05.016 PMC3730833 23809161

[55] Chen J Chen ZJ. PtdIns4P on dispersed trans-golgi network mediates NLRP3 inflammasome activation. Nature (2018) 564(7734):71–6. doi: 10.1038/s41586-018-0761-3 PMC9402428 30487600

[56] Vajjhala PR Mirams RE Hill JM. Multiple binding sites on the pyrin domain of ASC protein allow self-association and interaction with NLRP3 protein. J Biol Chem (2012) 287(50):41732–43. doi: 10.1074/jbc.M112.381228 PMC3516722 23066025

[57] He Y Zeng MY Yang D Motro B Núñez G. NEK7 is an essential mediator of NLRP3 activation downstream of potassium efflux. Nature (2016) 530(7590):354–7. doi: 10.1038/nature16959 PMC4810788 26814970

[58] Man SM Kanneganti TD. Regulation of inflammasome activation. Immunol Rev (2015) 265(1):6–21. doi: 10.1111/imr.12296 25879280 PMC4400844

[59] Liu X Zhang Z Ruan J Pan Y Magupalli VG Wu H. Inflammasome-activated gasdermin d causes pyroptosis by forming membrane pores. Nature (2016) 535(7610):153–8. doi: 10.1038/nature18629 PMC5539988 27383986

[60] Zheng D Liwinski T Elinav E. Inflammasome activation and regulation: toward a better understanding of complex mechanisms. Cell Discovery (2020) 6(1):36. doi: 10.1038/s41421-019-0135-5 32550001 PMC7280307

[61] Kayagaki N Warming S Lamkanfi M Walle LV Louie S Dong J. Non-canonical inflammasome activation targets caspase-11. Nature (2011) 479(7371):117–21. doi: 10.1038/nature10558 22002608

[62] Viganò E Diamond CE Spreafico R Balachander A Sobota RM Mortellaro A. Human caspase-4 and caspase-5 regulate the one-step non-canonical inflammasome activation in monocytes. Nat Commun (2015) 6(1):8761. doi: 10.1038/ncomms9761 26508369 PMC4640152

[63] Shi J Zhao Y Wang Y Gao W Ding J Li P. Inflammatory caspases are innate immune receptors for intracellular LPS. Nature (2014) 514(7521):187–92. doi: 10.1038/nature13683 25119034

[64] Sanders MG Parsons MJ Howard AGA Liu J Fassio SR Martinez JA. Single-cell imaging of inflammatory caspase dimerization reveals differential recruitment to inflammasomes. Cell Death Dis (2015) 6(7):e1813. doi: 10.1038/cddis.2015.186 26158519 PMC4650733

[65] Baker PJ Boucher D Bierschenk D Tebartz C Whitney PG D'Silva DB. NLRP3 inflammasome activation downstream of cytoplasmic LPS recognition by both caspase-4 and caspase-5. Eur J Immunol (2015) 45(10):2918–26. doi: 10.1002/eji.201545655 26173988

[66] Kayagaki N Stowe IB Lee BL O’Rourke K Anderson K Warming S. Caspase-11 cleaves gasdermin d for non-canonical inflammasome signalling. Nature (2015) 526(7575):666–71. doi: 10.1038/nature15541 26375259

[67] Piccini A Carta S Tassi S Lasiglié D Fossati G Rubartelli A. ATP is released by monocytes stimulated with pathogen-sensing receptor ligands and induces IL-1β and IL-18 secretion in an autocrine way. Proc Natl Acad Sci (2008) 105(23):8067–72. doi: 10.1073/pnas.0709684105 PMC2430360 18523012

[68] Gaidt MM Ebert TS Chauhan D Schmidt T Schmid-Burgk JL Rapino F. Human monocytes engage an alternative inflammasome pathway. Immunity (2016) 44(4):833–46. doi: 10.1016/j.immuni.2016.01.012 27037191

[69] Gaidt MM Hornung V. Alternative inflammasome activation enables IL-1β release from living cells. Curr Opin Immunol (2017) 44:7–13. doi: 10.1016/j.coi.2016.10.007 27842238 PMC5894802

[70] Peeters PM Perkins TN Wouters EF Mossman BT Reynaert NL. Silica induces NLRP3 inflammasome activation in human lung epithelial cells. Particle fibre Toxicol (2013) 10(1):1–11. doi: 10.1186/1743-8977-10-3 PMC3607900 23402370

[71] Song-Zhao GX Srinivasan N Pott J Baban D Frankel G Maloy KJ. Nlrp3 activation in the intestinal epithelium protects against a mucosal pathogen. Mucosal Immunol (2014) 7(4):763–74. doi: 10.1038/mi.2013.94 PMC4048180 24280937

[72] Klasson M Lindberg M Westberg H Bryngelsson IL Tuerxun K Persson A. Dermal exposure to cobalt studied *in vitro* in keratinocytes–effects of cobalt exposure on inflammasome activated cytokines, and mRNA response. Biomarkers (2021) 26(8):674–84. doi: 10.1080/1354750X.2021.1975823 34496682

[73] Duewell P Kono H Rayner KJ Sirois CM Vladimer G Bauernfeind FG. NLRP3 inflammasomes are required for atherogenesis and activated by cholesterol crystals. Nature (2010) 464(7293):1357–61. doi: 10.1038/nature08938 PMC2946640 20428172

[74] Halle A Hornung V Petzold GC Stewart CR Monks BG Reinheckel T. The NALP3 inflammasome is involved in the innate immune response to amyloid-β. Nat Immunol (2008) 9(8):857–65. doi: 10.1038/ni.1636 PMC3101478 18604209

[75] Codolo G Plotegher N Pozzobon T Brucale M Tessari I Bubacco L. Triggering of inflammasome by aggregated α–synuclein, an inflammatory response in synucleinopathies. PloS One (2013) 8(1):e55375. doi: 10.1371/journal.pone.0055375 23383169 PMC3561263

[76] Tao X Wan X Wu D Song E Song Y. A tandem activation of NLRP3 inflammasome induced by copper oxide nanoparticles and dissolved copper ion in J774A. 1 macrophage. J Hazard Mater (2021) 411:125134. doi: 10.1016/j.jhazmat.2021.125134 33485222

[77] Palomaki J Valimaki E Sund J Vippola M Clausen PA Jensen KA. Long, needle-like carbon nanotubes and asbestos activate the NLRP3 inflammasome through a similar mechanism. ACS nano (2011) 5(9):6861–70. doi: 10.1021/nn200595c 21800904

[78] Hari A Zhang Y Tu Z Detampel P Stenner M Ganguly A. Activation of NLRP3 inflammasome by crystalline structures *via* cell surface contact. Sci Rep (2014) 4(1):7281. doi: 10.1038/srep07281 25445147 PMC4250918

[79] Alijagic A Engwall M Särndahl E Karlsson H Hedbrant A Andersson L. Particle safety assessment in additive manufacturing: From exposure risks to advanced toxicology testing. Front Toxicol (2022) 4. doi: 10.3389/ftox.2022.836447 PMC9081788 35548681

[80] Chevriaux A Pilot T Derangère V Simonin H Martine P Chalmin F. Cathepsin b is required for NLRP3 inflammasome activation in macrophages, through NLRP3 interaction. Front Cell Dev Biol (2020) 8:167. doi: 10.3389/fcell.2020.00167 32328491 PMC7162607

[81] Dominic A Le NT Takahashi M. Loop between NLRP3 inflammasome and reactive oxygen species. Antioxid Redox Signaling (2022) 36(10):784–96. doi: 10.1089/ars.2020.8257 34538111

[82] Campden RI Zhang Y. The role of lysosomal cysteine cathepsins in NLRP3 inflammasome activation. Arch Biochem biophys (2019) 670:32–42. doi: 10.1016/j.abb.2019.02.015 30807742

[83] Morishige T Yoshioka Y Inakura H Tanabe A Yao X Narimatsu S. The effect of surface modification of amorphous silica particles on NLRP3 inflammasome mediated IL-1β production, ROS production and endosomal rupture. Biomaterials (2010) 31(26):6833–42. doi: 10.1016/j.biomaterials.2010.05.036 20561679

[84] Sun B Wang X Ji Z Li R Xia T. NLRP3 inflammasome activation induced by engineered nanomaterials. Small (2013) 9(9-10):1595–607. doi: 10.1002/smll.201201962 PMC4056676 23180683

[85] Sayan M Mossman BT. The NLRP3 inflammasome in pathogenic particle and fibre-associated lung inflammation and diseases. Particle fibre Toxicol (2015) 13:1–15. doi: 10.1186/s12989-016-0162-4 PMC5029018 27650313

[86] Wu R Högberg J Adner M Ramos-Ramírez P Stenius U Zheng H. Crystalline silica particles cause rapid NLRP3-dependent mitochondrial depolarization and DNA damage in airway epithelial cells. Particle Fibre Toxicol (2020) 17:1–20. doi: 10.1186/s12989-020-00370-2 PMC7418441 32778128

[87] Bauernfeind F Bartok E Rieger A Franchi L Núñez G Hornung V. Cutting edge: reactive oxygen species inhibitors block priming, but not activation, of the NLRP3 inflammasome. J Immunol (2011) 187(2):613–7. doi: 10.4049/jimmunol.1100613 PMC3131480 21677136

[88] Liu W Yin Y Zhou Z He M Dai Y. OxLDL-induced IL-1beta secretion promoting foam cells formation was mainly *via* CD36 mediated ROS production leading to NLRP3 inflammasome activation. Inflammation Res (2014) 63:33–43. doi: 10.1007/s00011-013-0667-3 24121974

[89] Lunov O Syrovets T Loos C Nienhaus GU Mailänder V Landfester K. Amino-functionalized polystyrene nanoparticles activate the NLRP3 inflammasome in human macrophages. ACS nano (2011) 5(12):9648–57. doi: 10.1021/nn203596e 22111911

[90] Busch M Bredeck G Waag F Rahimi K Ramachandran H Bessel T. Assessing the NLRP3 inflammasome activating potential of a Large panel of micro-and nanoplastics in THP-1 cells. Biomolecules (2022) 12(8):1095. doi: 10.3390/biom12081095 36008988 PMC9406042

[91] Caputi S Diomede F Lanuti P Marconi GD Di Carlo P Sinjari B. Microplastics affect the inflammation pathway in human gingival fibroblasts: A study in the Adriatic Sea. Int J Environ Res Public Health (2022) 19(13):7782. doi: 10.3390/ijerph19137782 35805437 PMC9266176

[92] Chen YC Chen KF Lin KYA Chen JK Jiang XY Lin CH. The nephrotoxic potential of polystyrene microplastics at realistic environmental concentrations. J Hazard Mater (2022) 427:127871. doi: 10.1016/j.jhazmat.2021.127871 34862106

[93] Wu Y Yao Y Bai H Shimizu K Li R Zhang C. Investigation of pulmonary toxicity evaluation on mice exposed to polystyrene nanoplastics: The potential protective role of the antioxidant n-acetylcysteine. Sci Total Environ (2023) 855:158851. doi: 10.1016/j.scitotenv.2022.158851 36155047

[94] Chi Q Xu T He Y Li Z Tang X Fan X. Polystyrene nanoparticle exposure supports ROS-NLRP3 axis-dependent DNA-NET to promote liver inflammation. J Hazard Mater (2022) 439:129502. doi: 10.1016/j.jhazmat.2022.129502 35868089

[95] Hou J Lei Z Cui L Hou Y Yang L An R. Polystyrene microplastics lead to pyroptosis and apoptosis of ovarian granulosa cells *via* NLRP3/Caspase-1 signaling pathway in rats. Ecotoxicol Environ Saf (2021) 212:112012. doi: 10.1016/j.ecoenv.2021.112012 33550074

[96] Wei J Wang X Liu Q Zhou N Zhu S Li Z. The impact of polystyrene microplastics on cardiomyocytes pyroptosis through NLRP3/Caspase-1 signaling pathway and oxidative stress in wistar rats. Environ Toxicol (2021) 36(5):935–44. doi: 10.1002/tox.23095 33404188

[97] Choi YJ Kim JE Lee SJ Gong JE Jin YJ Seo S. Inflammatory response in the mid colon of ICR mice treated with polystyrene microplastics for two weeks. Lab Anim Res (2021) 37(1):1–9. doi: 10.1186/s42826-021-00109-w 34809705 PMC8607556

[98] Mu Y Sun J Li Z Zhang W Liu Z Li C. Activation of pyroptosis and ferroptosis is involved in the hepatotoxicity induced by polystyrene microplastics in mice. Chemosphere (2022) 291:132944. doi: 10.1016/j.chemosphere.2021.132944 34793849

[99] Danso IK Woo JH Lee K. Pulmonary toxicity of polystyrene, polypropylene, and polyvinyl chloride microplastics in mice. Molecules (2022) 27(22):7926. doi: 10.3390/molecules27227926 36432032 PMC9694469

[100] Zhong G Rao G Tang L Wu S Tang Z Huang R. Combined effect of arsenic and polystyrene-nanoplastics at environmentally relevant concentrations in mice liver: Activation of apoptosis, pyroptosis and excessive autophagy. Chemosphere (2022) 300:134566. doi: 10.1016/j.chemosphere.2022.134566 35413363

[101] He Y Li Z Xu T Luo D Chi Q Zhang Y. Polystyrene nanoplastics deteriorate LPS-modulated duodenal permeability and inflammation in mice *via* ROS drived-NF-κB/NLRP3 pathway. Chemosphere (2022) 307:135662. doi: 10.1016/j.chemosphere.2022.135662 35830933

[102] Nikolic S Gazdic-Jankovic M Rosic G Miletic-Kovacevic M Jovicic N Nestorovic N. Orally administered fluorescent nanosized polystyrene particles affect cell viability, hormonal and inflammatory profile, and behavior in treated mice. Environ pollut (2022) 305:119206. doi: 10.1016/j.envpol.2022.119206 35405220

[103] Zhang Y Yin K Wang D Wang Y Lu H Zhao H. Polystyrene microplastics-induced cardiotoxicity in chickens *via* the ROS-driven NF-κB-NLRP3-GSDMD and AMPK-PGC-1α axes. Sci Total Environ (2022) 840:156727. doi: 10.1016/j.scitotenv.2022.156727 35714743

[104] Yin K Lu H Zhang Y Hou L Meng X Li J. Secondary brain injury after polystyrene microplastic-induced intracerebral hemorrhage is associated with inflammation and pyroptosis. Chemico-Biol Interact (2022) 367:110180. doi: 10.1016/j.cbi.2022.110180 36113630

[105] Cao J Xu R Wang F Geng Y Xu T Zhu M. Polyethylene microplastics trigger cell apoptosis and inflammation *via* inducing oxidative stress and activation of the NLRP3 inflammasome in carp gills. Fish Shellfish Immunol (2023) 132:108470. doi: 10.1016/j.fsi.2022.108470 36470402

[106] Swartzwelter BJ Mayall C Alijagic A Barbero F Ferrari E Hernadi S. Cross-species comparisons of nanoparticle interactions with innate immune systems: A methodological review. Nanomaterials (2021) 11(6):1528. doi: 10.3390/nano11061528 34207693 PMC8230276

[107] Yee MSL Hii LW Looi CK Lim WM Wong SF Kok YY. Impact of microplastics and nanoplastics on human health. Nanomaterials (2021) 11(2):496. doi: 10.3390/nano11020496 33669327 PMC7920297

[108] Alijagic A Scherbak N Kotlyar O Karlsson P Wang X Odnevall I. A novel nanosafety approach using cell painting, metabolomics, and lipidomics captures the cellular and molecular phenotypes induced by the unintentionally formed metal-based (Nano) particles. Cells (2023) 12(2):281. doi: 10.3390/cells12020281 36672217 PMC9856453

[109] Yang W Li Y Boraschi D. Association between microorganisms and microplastics: How does it change the host–pathogen interaction and subsequent immune response? Int J Mol Sci (2023) 24(4):4065. doi: 10.3390/ijms24044065 36835476 PMC9963316

